# Patient specific instrumentation for open Latarjet procedure. Technique, accuracy, and short-term outcome. A prospective case series

**DOI:** 10.1016/j.xrrt.2025.05.005

**Published:** 2025-05-29

**Authors:** Emmie A.M. van den Elzen, Kshitij Gupta, Esther Janssen, Berend Geelen, Maichel Gommans, Bruno Gobbato, Okke Lambers Heerspink, Freek Hollman

**Affiliations:** a Physician Clinical Investigator, Faculty of Health, Medicine and Life Sciences, Maastricht University, Maastricht, Limburg, the Netherlands; b Department of Orthopaedics, All India Institute of Medical Sciences, Rishikesh, Uttarakhand, India; c Department of Orthopaedic Surgery, VieCuri Medical Centre, Venlo, Limburg, the Netherlands; d Research Institute for Health Sciences, IQ Health, Radboud University Medical Center, Nijmegen, Gelderland, the Netherlands; e School of Allied Health, HAN University of Applied Sciences, Nijmegen, Gelderland, the Netherlands; f Technical Medical Department, VieCuri Medical Centre, Venlo, Limburg, the Netherlands; g IDOMED Jaraguá Medical University, Jaraguá do Sul, Santa Catarina, Brazil; h Department of Orthopaedic Surgery, Maastricht University Medical Center+, Maastricht, Limburg, the Netherlands

**Keywords:** Shoulder instability, Latarjet procedure, Patient-specific instrumentation (PSI), 3D technology, Coracoid graft positioning, Graft consolidation

## Abstract

**Background:**

The Latarjet procedure is a common technique to address anteroinferior shoulder instability. However, inadequate positioning of the coracoid bone graft may lead to persistent instability or early arthritis, with complication rates described up to 15% in open procedures. Adequate bone-block positioning tailored to patients’ scapular morphology may reduce these complications. This study aims to optimize the Patient-Specific Instrumentation (PSI) Latarjet procedure by detailing the technique and assessing the accuracy of graft positioning through a comparison of the digitally planned and actual surgical outcomes. It is hypothesized that patient-specific, 3D-printed guides will enhance coracoid graft accuracy in the open Latarjet procedure.

**Methods:**

Between January and May 2024, 5 patients underwent the open PSI Latarjet procedure. Using computer aided design software, the procedure was digitally planned, followed by the design and production of PSI drill guides utilized in a standard double screw technique. Patients were immobilized for three weeks with radiological evaluations at 6 weeks postoperatively.

**Results:**

All patients were included in the analysis. Compared to digitally planning, the median mediolateral displacement was 0.01 mm medially (range 1.8 mm medially-1.7 mm laterally), and the median craniocaudal displacement was 0.97 mm caudally (range 5.16 mm caudally-5.58 mm cranially), from what was digitally planned. Modifications to the PSI guides were made over time to improve accuracy, though one screw was malpositioned in a single case. However, all grafts showed evidence of graft healing within 6 weeks.

**Conclusion:**

The open PSI Latarjet procedure is safe and reliable, showing accurate graft positioning at six weeks postsurgery. We believe that 3D printing marks a significant advancement for surgeons, allowing a shift from conventional instruments to innovative, customized solutions.

The Latarjet procedure is a common surgical procedure to address anteroinferior shoulder instability, particularly in cases of significant glenoid bone loss.^20^ Despite advancements in graft positioning since its introduction in 1954,^16^^,^^18^^,^^20^ inadequate graft positioning and implant-related complications, such as misalignment or overtightening of the screws, remain significant concerns, increasing the risk of postoperative complications, including osteoarthritis.^16^

The patient-specific instrumentation (PSI) Latarjet procedure aims to improve graft placement accuracy through 3D-printed surgical guides.^24^^,^^30^ This approach enables surgeons to evaluate patient-specific anatomy, plan critical surgical steps, and translate this preoperative plan into precise intraoperative execution.^16^

Although 3D patient-specific guides have been effectively applied in shoulder arthroplasty,^24^ its application in Latarjet surgery represents a new frontier aimed at improving outcomes. Whereas clinical data on PSI in open Latarjet are limited, initial findings suggest improved graft accuracy, reduced mechanical stress, and fewer graft and screw malposition complications.^16^^,^^24^

This case series aims to optimize the PSI Latarjet procedure by detailing the technique and assessing the accuracy of graft and screw positioning through comparing the digital performance of the procedure with the open PSI Latarjet procedure. It is hypothesized that the use of patient-specific 3D-printed guides in the open Latarjet procedure may enhance the accuracy of coracoid graft positioning.

## Materials and methods

### Study design and patient characteristics

This prospective case study included 5 patients who underwent open PSI Latarjet procedure between January 2024 and May 2024 at VieCuri Medical Centre, Venlo, The Netherlands. Patients' clinical history was obtained, emphasizing the etiology of the anteroinferior shoulder instability, frequency of shoulder dislocations, and their level of participation in either contact or non-contact sports. A comprehensive clinical examination was performed. Preoperative computed tomography (CT) scanning was conducted in all patients to assess the extent of glenoid bone loss, using the best-fit circle, and to identify any associated Hill-Sachs lesion. A threshold of 20% glenoid bone loss was set for proceeding with the Latarjet procedure to restore shoulder stability and function. Key parameters assessed included the percentage of glenoid bone loss, along with associated cuff and SLAP lesions. This study received approval from the Institution's Ethics Committee, ensuring adherence to ethical standards in patient evaluation and treatment (2020-2385).

Preoperative CT scanning was conducted in all patients to assess the extent of glenoid bone loss, using the best-fit circle method, and to identify any associated Hill-Sachs lesions. A threshold of 20% glenoid bone loss was set for proceeding with the Latarjet procedure to restore shoulder stability and function.

### Preoperative planning and surgical technique

**Figure 1:**
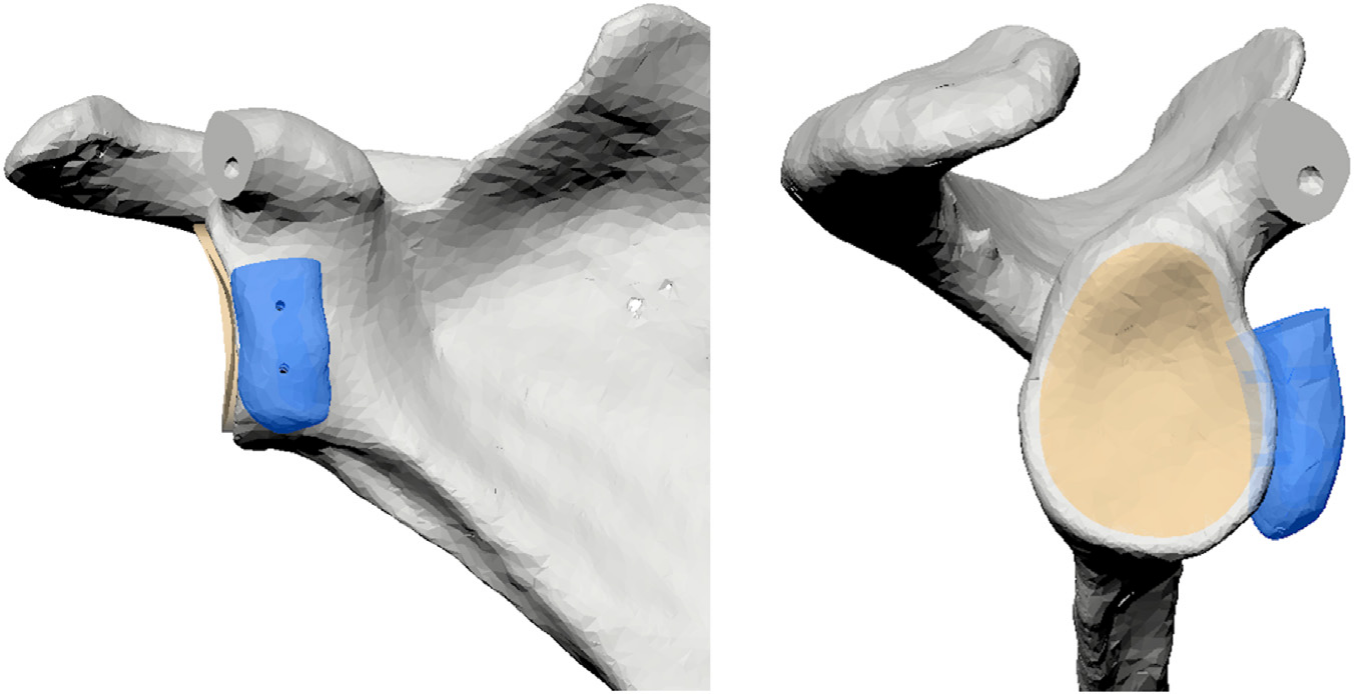
Preoperative segmentation coracoid graft.

**Figure 2:**
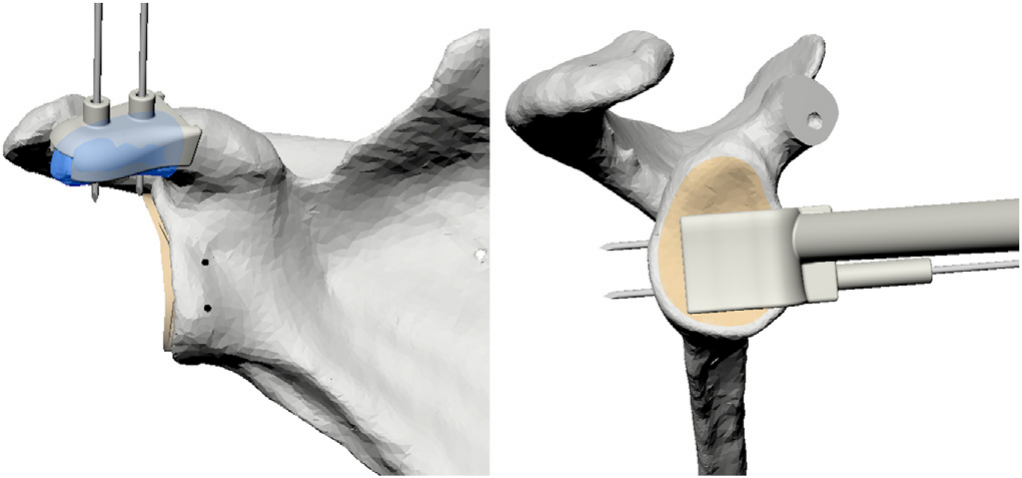
Preoperative positioning coracoid guide and glenoid guide.

Using the DICOM (Digital Communications in Medicine) files of the preoperative CT-scan a three-dimensional computed tomography (3D CT) reconstruction of the whole scapula was performed to outline the glenoid and coracoid processus morphology. The coracoid osteotomy was digitally simulated using Autodesk Fusion software (Autodesk Fusion 360, version 2.0.20754 x86_64; Autodesk, San Francisco, California, USA), which is a software used for professional product design and production. The cut was planned perpendicular to the longest axis of the coracoid at the inferior cortical inflection point, known as the "knee" of the coracoid process. The coracoid graft was then digitally positioned on the anteroinferior border of the glenoid to address the glenoid bone loss, as demonstrated in Figure 1 and Supplementary Video S1. Then, a patient-specific mold around the superior surface of the coracoid graft was designed, perfectly adapting the shape of the coracoid. The mold contained 2 1.25 mm guide wire cylinders, to allow for the passage of the guide wires through the graft, as demonstrated in Figure 2.

Similar to the mold of the coracoid graft, a patient-specific mold around the anterior glenoid rim and anterior surface was digitally designed, with 1.25 mm guide wire cylinders, to allow for the passage of the K-wires (Fig. 2). Final approval of the molds required consensus between a medical technician (M.G.) and an orthopedic surgeon (F.H.). Following the digital design of the coracoid and glenoid templates, real-size 3D models were produced for the purpose of testing, validation, and preoperative education of the surgical team. Then, the definitive molds were printed by Oceanz 3D Printing (Oceanz 3D Printing, Ede, The Netherlands) and were sterilized by the department. After sterilization, the molds were packed and stored until use during surgery.

**Figure 3:**
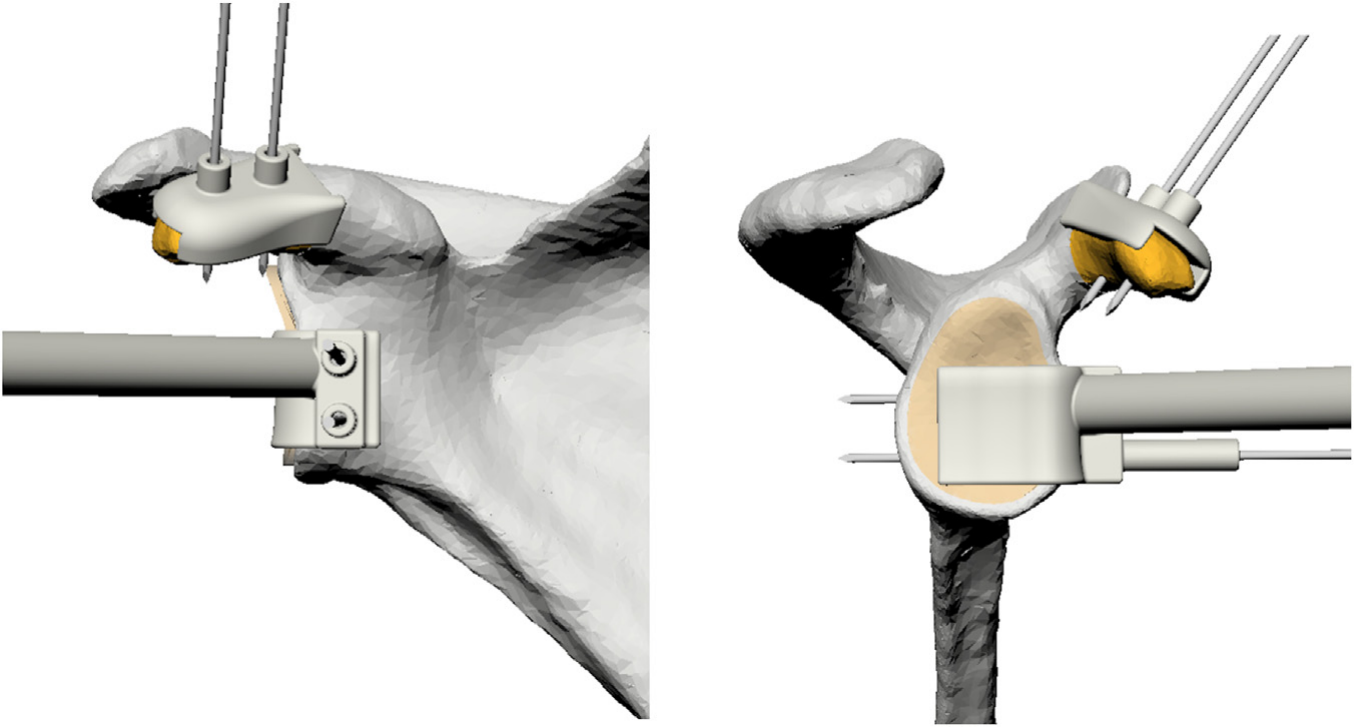
Coracoid guide and glenoid guide in situ.
(4) A subscapularis split was made to expose the underlying joint capsule. A capsulotomy was performed. The anterior-inferior glenoid was minimally decorticated, as the use of PSI guides reduced the need for surface alteration. The glenoid PSI guide was positioned in the preestablished position, and 2 K-wires were positioned corresponding with the position of the drill holes in the coracoid process (Fig. 3).
(5) The coracoid graft was then positioned over the previously inserted K-wires, and its placement was subsequently evaluated to ensure that no overhang was present. The bone graft was secured utilizing 2 cannulated screws and a wedge plate (Arthrex Inc., Naples, Florida, USA). The principles of the open PSI Latarjet procedure are illustrated in Supplementary Video S1.

All double-screw PSI Latarjet procedures were performed by a single orthopedic surgeon (F.H.). The process included the following steps:

(1) A deltopectoral approach was performed.
(2) The coracoacromial ligament and coracohumeral were ligated from its attachment on the coracoid. The medial aspect of the coracoid process was fully exposed by releasing the pectoralis minor attachment.
(3) The coracoid PSI guide was then positioned, and 2 K-wires were drilled through the pre-established positions on the guide (Fig. 3). Through the posterior located slot, the superior aspect of the cortical bone was cut using an oscillating saw. The guide was removed, and the cut was completed to harvest the coracoid process.

After the open Latarjet procedure, the affected arm was immobilized in a sling for a period of 3 weeks. After these 3 weeks, patients were gradually encouraged to resume daily activities, with movements permitted up to 90 degrees of abduction and restricted external rotation. At 6 weeks, upon confirmation of complete graft consolidation by CT scanning, all movement restrictions were lifted. Patients were permitted to return to contact sports activities after 6 months, contingent upon the full recovery of shoulder function.

### PSI guide modifications

As the use of 3D-printed PSI guides for Latarjet procedures is still emerging, modifications to the mold design were made continuously throughout the period of performing the surgeries. With each procedure, feedback from the orthopedic surgeon (F.H.), along with observations of intraoperative performance and postoperative outcomes, was systematically reviewed with the medical technician (M.G.), and integrated into subsequent designs. This iterative process allowed for ongoing refinement of the PSI guides.

### Evaluation of graft positioning

Coracoid graft and superior screw accuracy was determined by comparing preoperative and 6-week postoperative 3D CT scans. By overlaying the scans, the Autodesk Fusion software allowed for precise measurement of the alignment and positioning of the bone graft and superior screw, assessing how closely the actual surgical outcomes matched the preoperative digital plan. A medical technician (M.G.) and the primary investigator (E.E.) manually established the sagittal plane as the initial reference point. Once defined, automated rotations of 90° were performed to the transverse and coronal planes. In the sagittal and transverse planes, the craniocaudal orientation and anteroposterior inclination of the superior screw were analyzed to assess its multidirectional alignment preoperatively versus postoperatively. Craniocaudal and mediolateral deviations were measured by calculating the angle between the planned and intraoperative screw positions, with larger angles indicating greater deviation from the intended positioning and, consequently, greater misalignment. The coronal plane was used to assess craniocaudal displacement and mediolateral overhang of the bone graft relative to the preoperative plan. The craniocaudal displacement was evaluated by measuring the distance from the center of the preoperatively planned bone graft to the center of the intraoperatively placed bone graft. The mediolateral overhang was evaluated by measuring the distance from the center of the preoperatively positioned bone graft to its lateral edge, which represented the glenoid rim, as the lateral edge was precisely delineated with the glenoid rim through preoperative imaging. A corresponding measurement was then taken from the center of the intraoperative bone graft to its lateral edge. The difference between these measurements indicated the mediolateral positional deviation. In cases 1-4, a 2.4 mm correction for glenoid cartilage thickness was applied to prevent lateralized graft fixation. Final angle measurements required the consensus of 2 investigators (E.E. & M.G.) before a definitive value was assigned.

### Evaluation of graft healing

Graft healing was radiologically assessed at 6 weeks postoperatively using CT scans to evaluate the early phase of bone integration at the graft-host interface. In this context, graft healing refers to the presence of trabecular continuity between the coracoid graft and the native glenoid, indicating the initiation of bone integration. This should be distinguished from graft consolidation, which represents a more advanced stage of healing. The assessment of graft healing was performed by an orthopedic surgeon (F.H.).

### Statistical analysis

Patient demographics, clinical outcomes, area of glenoid bone loss, graft dimensions, and angles of deviation from the preoperative plan were described using descriptive statistics. Dichotomous variables were reported as counts or values, while quantitative data were presented as medians with corresponding ranges. Statistical analysis was conducted using Microsoft Excel.

## Results

### Patient data

All 5 patients were male, with 2 having left-sided and 3 having right-sided shoulder involvement. The median age at the time of surgery was 23 (range 21-33) years, with a median body mass index of 23.46 (range 19.39-28.91). The median glenoid bone loss was 5%, ranging from 2-17% of the total glenoid surface area. All patients had a concomitant Hill-Sachs injury to the humeral head. Further details can be found in Table I.

**Table I:** Patient data.

Parameter	Value
Age*, yr, (median* ± *range)*	23 (21-33)
Gender, *male/female, n*	5/0
BMI, *kg*^*2*^*(median* ± *range)*	23.46 (19.39-28.91)
Shoulder side, *left/right, n*	2/3
Smoking, *n*	3
Contactsport, *n*∗	3
Glenoid bone loss *(percentage, median* ± *range)*	5 (2-17)
Hill Sachs, *n*	5
Cuff lesion, *n*	0
SLAP lesion, *n*	0

*BMI*, body mass index; *SLAP*, superior labrum anterior to posterior.

∗ At time of first luxation.

### Surgical details and recovery

No postoperative infection, axillary nerve injury, or vascular injury occurred in any patient during the 6-week follow-up. One out of 5 patients had mispositioning of the superior screw which was positioned chondrally based on the CT scanning at 6 weeks after surgery and was directly removed arthroscopically.

The design of the PSI guides underwent several refinements to enhance fit, usability, and accuracy. In total, 4 different coracoid guides and 4 different glenoid guides were utilized across all procedures. The major modifications included: 1) The inner surfaces of both guides were contoured to match the bone's anatomy, ensuring a precise fit. The outer parts of the templates were modified to adopt a more organic shape, aligning closely with the morphology of the coracoid and glenoid. 2) A cutout was incorporated into the coracoid template to accommodate the tendons attached to the coracoid process, addressing a significant limitation of the initial design where the attached tendons prevented proper positioning of the guide. 3) The length of the drill guides on both templates was shortened, as the extended length in the initial design did not impact the drilling trajectory but posed challenges in accurately positioning the guide against the glenoid. 4) The saw slot was removed from both templates, replaced with a recess featuring a flat surface for sawing. 5)

To account for the cartilage rim on the glenoid surface, a 2.4 mm notch was incorporated into the glenoid mold of the fifth case.^26^ These alterations ensured optimal alignment and effectively prevented excessive lateralization of graft fixation. A comprehensive overview of the adjustments and the specific molds used for each procedure is provided in the Supplementary Table S1.

### Coracoid graft positioning and healing

The mediolateral graft displacement demonstrated a median of 0.01 mm medially (range 1.8 mm medial to 1.7 mm lateral), compared to the digital plan. Of the 5 cases, 3 exhibited medial displacement and 2 showed lateral displacement. Craniocaudal positioning demonstrated a median deviation of 0.97 mm in the caudal direction (range 5.16 mm caudal to 5.58 mm cranial) from the preoperative plan. As shown in Table II, 3 of the 5 cases exhibited caudal displacement, while 2 demonstrated cranial displacement. The angular craniocaudal deviations ranged from 4.89° to 16.95°, while the mediolateral deviations varied from 2.56° to 20.75° (Table II). At 6 weeks postoperatively, all 5 bone grafts showed evidence of graft healing—defined as trabecular continuity between the graft and native glenoid—in both the inferior and superior aspects of the graft, without signs of full graft consolidation.

**Table II:** Coracoid bone graft positioning.

Patient	Shoulder side	Angular deviation sagittal plane (°)	Cranial/Caudal (mm)	Angular deviation transversal plane (°)	Lateral/Medial (mm)∗
Case 1	R	16.95	+1.04	14.03	+1.13
Case 2	L	11.36	+5.58	8.20	−0.01
Case 3	L	6.25	−3.17	2.56	−1.8
Case 4	R	4.89	−0.97	20.75	−1.66
Case 5	R	8.01	−5.16	20.14	+1.70

∗ Corrected for 2.4 mm cartilage on glenoid surface.

## Discussion

The Latarjet procedure is effective for managing anterior shoulder instability, but complication rates of 15-30% are reported.^9^^,^^13^ Graft malposition remains a key concern, with medial placement risking recurrent instability and lateral placement increasing joint contact forces and early osteoarthritis.^9^^,^^11^^,^^27^ This case series aimed to optimize the 3D printed PSI Latarjet procedure to improve graft positioning. The study assessed the accuracy of PSI by comparing the actual coracoid graft position with the preoperative planning. The median graft position was 0.01 mm medial and 0.97 mm caudal to the glenoid surface. Superior screw alignment showed craniocaudal deviations of 4.89°-16.95° and mediolateral deviations of 2.56°-20.75°. All grafts achieved trabecular continuity within 6 weeks.

Previously, commercially available drill guides demonstrated to improve graft positioning compared to freehand drilling. Klatte et al tested these commercially available drill guides in 5 cadaveric shoulders, noting their ability to ease graft positioning and prevent screw misplacement.^15^ Meyer et al reported accurate graft placement and good compression in 12 open Latarjet procedures using a drill guide, with all screws placed medially to the articular edge.^22^ A comparative study between the freehand technique and drill guide technique was done by Barth et al and found that parallel drill guides improved accuracy in graft placement but with inferior bone contact. While commercially available guides enhance accuracy, they lack adaptability for patient variability.

Regarding anatomical differences in size and shape of glenoid and coracoid described between populations and genders, we believe that a customized drill guide can lead to better graft and screw positioning.^1^^,^^4^ PSI guides offer an individualized approach, designed to match patient anatomy, potentially reducing surgical time and surgeon related error rates.^6^^,^^8^^,^^29^ In spine and knee surgeries, PSI has significantly reduced operative time.^5^^,^^7^^,^^21^^,^^28^ 3D-printed PSI has shown success in shoulder arthroplasty, with Gauci et al demonstrating accurate glenoid placement in 17 cases.^8^ Studies by Elsheikh et al^6^ and Yung et al^29^ reported slightly shorter, though not statistically significant, surgical times. Similar benefits are expected in the Latarjet procedure.

This study used 3D-printed PSI guides to assess graft and screw positioning accuracy by comparing digital planning with the open PSI Latarjet procedure. Median graft placement was 0.01 mm medial (range: 1.8 mm medial to 1.7 mm lateral), within the acceptable 3 mm lateral to 5 mm medial range.^2^^,^^20^^,^^26^ Screw angulations showed greater deviations from the preoperative plan. Much emphasis has been put on screw parallelism with the glenoid surface, as higher angulations increase complication risks.^14^^,^^19^ Hsu et al^12^ showed that graft displacement remained unaffected up to 15° angulation but recommended avoiding angles >30°. In this study, screw angulations ranged from 2.56° to 20.75°, all below the 30° threshold. Pseudoarthrosis occurs in 1.5-9% of cases^3^^,^^23^; in our series, one superior screw was malpositioned and removed arthroscopically after graft healing. The surgical mold initially did not account for cartilage thickness, prompting a 2.4 mm adjustment in the fifth case to improve graft and screw positioning.

This study has several limitations. As a small series, its findings are not generalizable. However, this is the first study to evaluate PSI-guided Latarjet outcomes. Secondly, initial graft and superior screw planning was done manually, with consensus between 2 investigators to reduce interobserver variability, though some may remain. Moreover, the ideal graft and screw positioning has not been clearly defined in the literature. The study compares the actual graft positioning with the preoperative plan, which was based on discretion of the orthopedic surgeon. The 6-week follow-up showed evidence of graft healing in all cases, but long-term outcomes such as recurrent instability or osteoarthritis remain unknown. Lastly, continued mold adjustments suggest the fifth version may still be suboptimal. A larger cohort is needed for further refinement and validation. Strengths of the study include 3D analysis for more accurate evaluation, and consistent involvement of the same surgeon, technician, and investigator throughout the study, minimizing procedural variability.

This is a first series using PSI instruments for open Latarjet technique, to the best of our knowledge. Future research should focus on the long-term outcomes of the PSI Latarjet procedure, particularly regarding graft positioning and implant-related complications. The introduction of the Latarjet procedure by Lafosse^17^ marked a shift toward minimally invasive management of glenoid bone loss in antero-inferior instability. While arthroscopic technique provides us with a clear view of the glenoid surface allowing a more accurate placement of the graft,^6^ comparative studies have found the graft positioning to be inferior as compared to the open technique.^25^^,^^26^ Moreover, arthroscopic procedures are typically more time-consuming than open surgery.^10^ PSI-guided techniques may address these limitations by improving graft placement accuracy and reducing the technical demands of the arthroscopic approach. Adaptation of PSI drill guides for arthroscopic use could potentially shorten the learning curve and enhance procedural consistency.

## Conclusion

Current study highlights the steps in developing PSI drill guides for Latarjet procedure. The PSI guides were modified over time to improve accuracy and performance. Despite this, malpositioning of one screw occurred in a single case. The use of 3D printed guides resulted in accurate graft positioning and graft healing. Long term comparative studies with larger sample sizes are needed to establish the validity of PSI guides in this procedure. Though, the study stems its potential use in Latarjet procedure to achieve good radiologic and functional outcome.

## Disclaimers:

Funding: No funding was disclosed by the authors.

Conflicts of interest: Okke Lambers Heerspink reports grants from 10.13039/100007307Arthrex, Inc. as paid consultant and research support. All the other authors, their immediate families, and any research foundation with which they are affiliated have not received any financial payments or other benefits from any commercial entity related to the subject of this article.
