# Supplementary material for: Patient specific instrumentation for open Latarjet procedure. Technique, accuracy, and short-term outcome. A prospective case series
Source: JSES Rev Rep Tech. 2025 May 29;5(4):1001–6. doi: 10.1016/j.xrrt.2025.05.005 (PMC12573450; doi:10.1016/j.xrrt.2025.05.005)
Supplement: Supplementary Table S1 [file mmc2.docx]

| **Case** | **Coracoid Guide** | **Glenoid Guide** | **Sagittal and frontal view scapula** | **Description** |
| --- | --- | --- | --- | --- |
| Case 1 (R) | 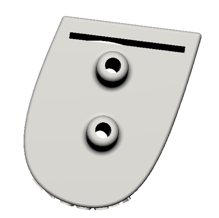 | 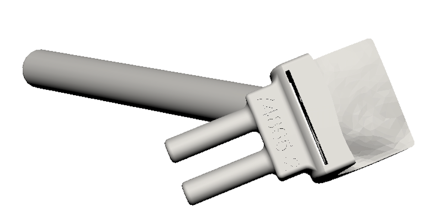 | 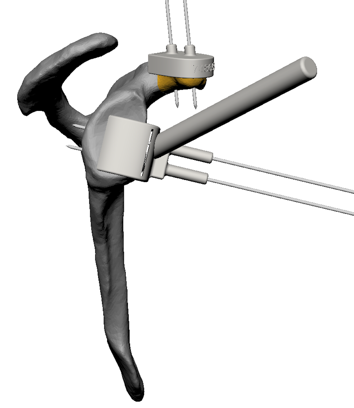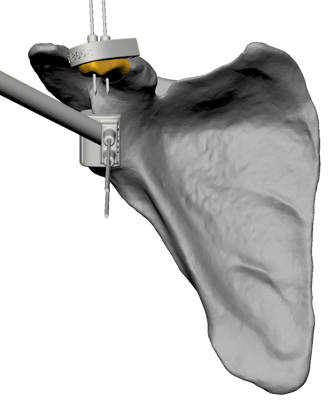 | This design is based on earlier prototypes that were not clinically applied. Characteristics in guides used for case 1:   - A drill guide is present on the glenoid template, as well as a smaller guide on the coracoid template, indicating the direction for drilling K-wires and placing cannulated screws. - Saw slots are incorporated, indicating the precise location for bone cutting. - Inner part of both guides is contoured to match the bone's shape, ensuring a perfect fit. - The glenoid guide includes a handle angled at 45 degrees upwards, allowing the surgeon to apply adequate pressure while approaching the glenoid from a superior direction during the procedure. - The template was still relatively bulky, which made accurate positioning on the glenoid challenging. |
|  | 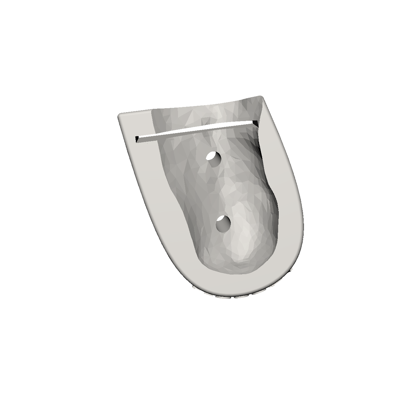 | 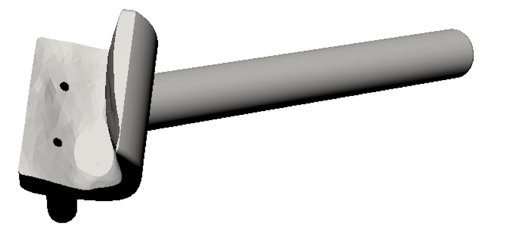 |  |  |
|  | 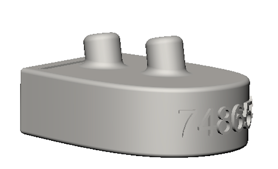 | 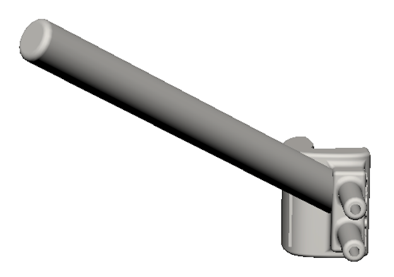 |  |  |

| Case 2 (L) | 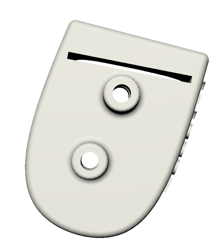 | 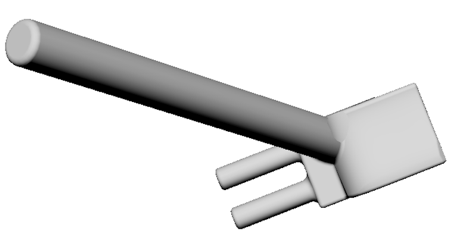 | 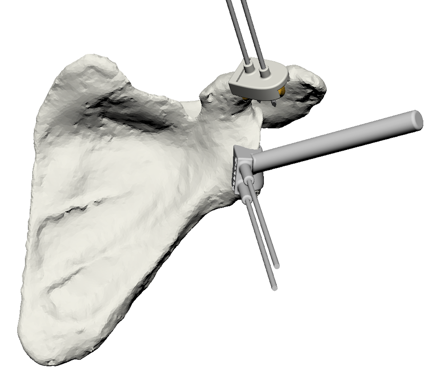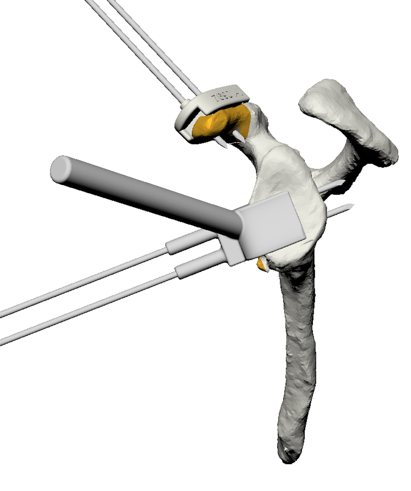 | Innovative aspect(s):   - Incorporating a cutout in the coracoid template for the attached tendons. The main issue with the initial design was the tendon attached to the coracoid process, which prevented the guide from being properly positioned on the coracoid.   Characteristics in guides used for case 2:   - Drill guide on the coracoid and glenoid template. - Saw slots on the coracoid and glenoid template. - Inner part of templates contoured to match the bone’s shape. - Glenoid template including a handle positioned 45 degrees upwards. - Bulky design. - Cutout coracoid template for attached tendons. |
| --- | --- | --- | --- | --- |
|  | 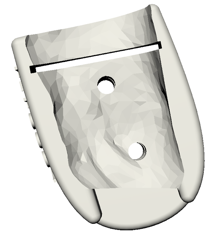 | 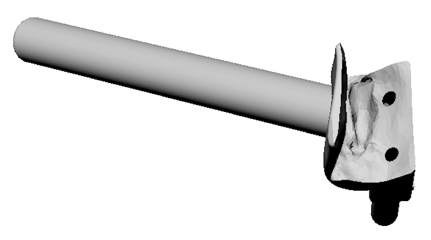 |  |  |
|  | 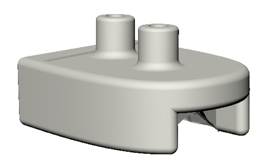 | 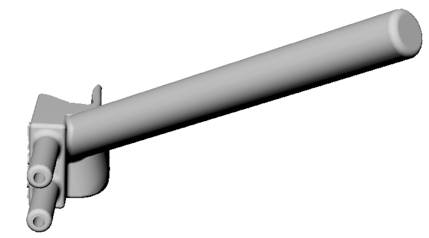 |  |  |
| Case 3 (L) | 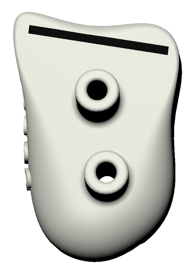 | 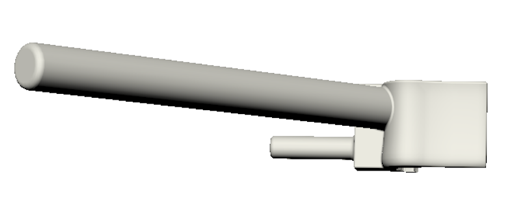 | 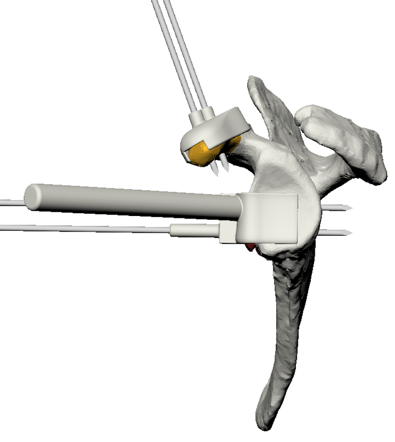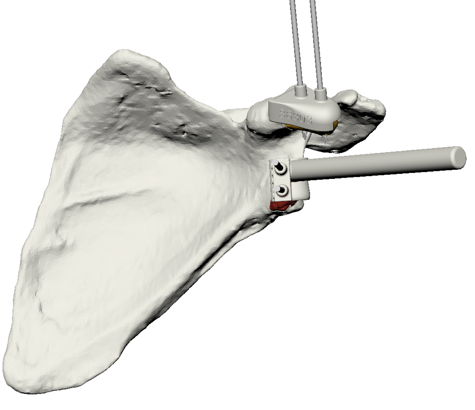 | Innovative aspect(s):   - Shorter drill guides on glenoid template, as their extended length did not affect the drilling direction but made it more challenging to position the guide accurately against the glenoid. - The saw slot was removed from the glenoid template, due to the slimmer design and its limited practical utility during surgery - Handle now extends perpendicular from the guide, as 45 degrees angle obstructed the surgical view. - Less bulky design, more aligned with the shape of coracoid as well as glenoid.   Characteristics in guides used for case 3:   - Short drill guide on both templates. - Saw slot only presented on coracoid template. - Inner part of templates contoured to match the bone’s shape. - Handle of glenoid template perpendicular from the guide. - Outer part of templates more organic with the shape of coracoid as well as glenoid. - Cutout coracoid template for attached tendons. |
|  | 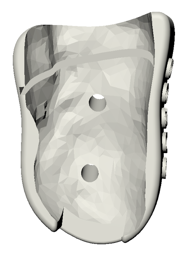 | 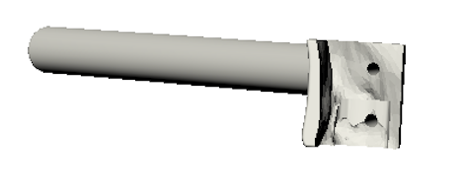 |  |  |
|  | 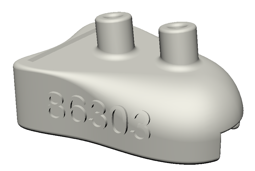 | 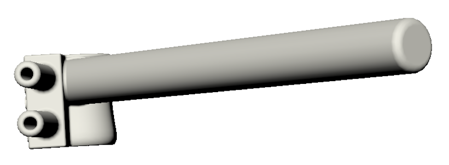 |  |  |
| Case 4 (R) | 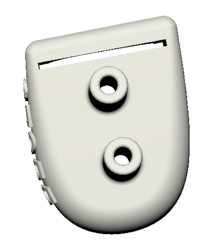 | 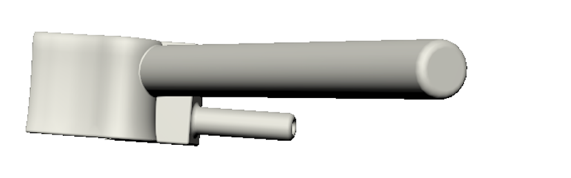 | 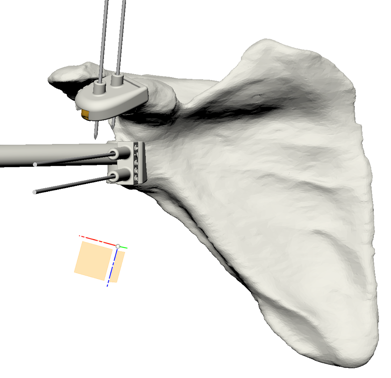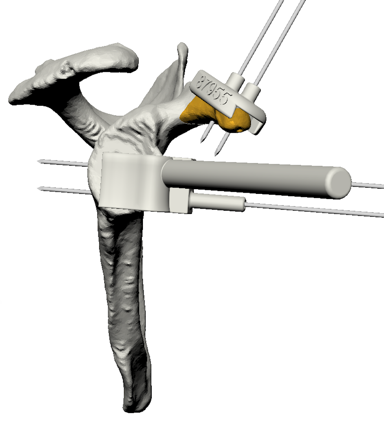 | No changes regarding the coracoid and glenoid templates were made between case 3 and 4. So, characteristics in guides used for case 4:   - Short drill guides on both templates. - Saw slot only presented on coracoid template. - Inner part of templates contoured to match the bone’s shape. - Handle of glenoid template perpendicular from the guide. - Outer part of templates more organic with the shape of coracoid as well as glenoid. - Cutout coracoid template for attached tendons. |
|  | 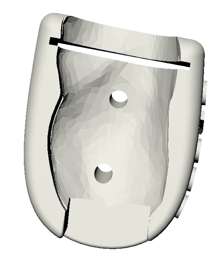 | 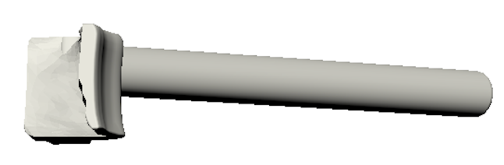 |  |  |
|  | 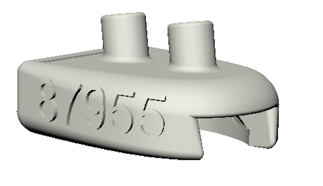 | 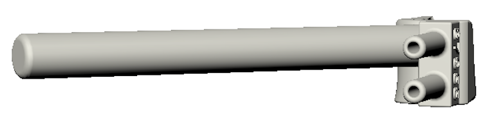 |  |  |
| Case 5 (R) | 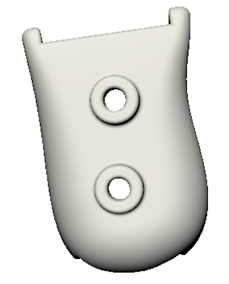 | 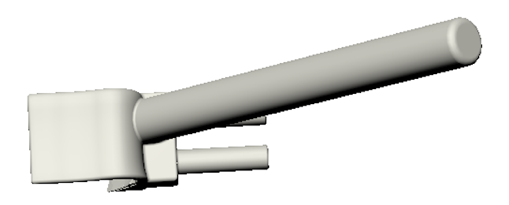 | 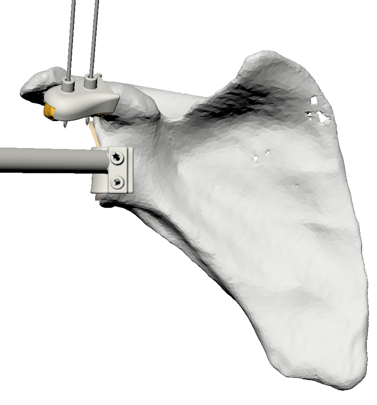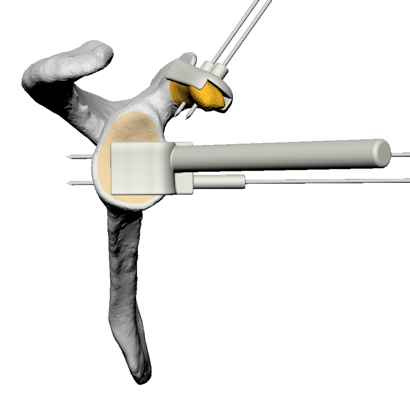 | Innovative aspect(s):   - The contact surface of the glenoid template has been shortened to half the size of the glenoid surface, with a 2.4 mm notch, reflecting the cartilage rim on the glenoid surface. - Further adapting to the anatomy, resulting in a more organic shape of coracoid template. - The saw slot has been removed from the coracoid template; however, a recess has been included with a flat surface for sawing, ensuring that the template does not become unnecessarily larger.   Characteristics in guides used for case 5:   - Short drill guides on both templates. - No saw slot presented on both templates, only a recess within the coracoid template. - Inner part of templates contoured to match the bone’s shape. - Handle of Glenoid template perpendicular from the guide. - Outer part even more organic with the shape of the coracoid. - Cutout coracoid template for attached tendons. - Shorter contact surface of the glenoid template, with a 2.4 mm notch. |
|  | 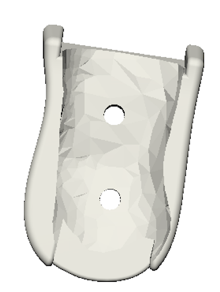 | 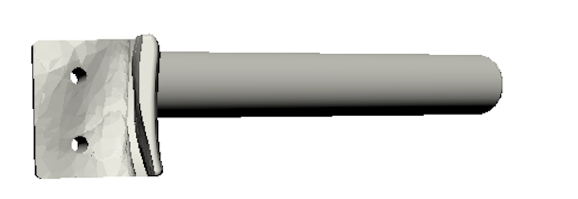 |  |  |
|  | 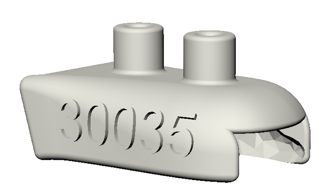 | 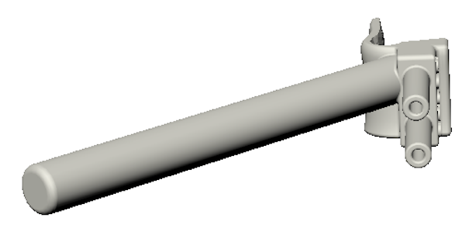 |  |  |
